# Acceleration of type 1 diabetes onset during the COVID-19 pandemic

**DOI:** 10.3389/fendo.2026.1841034

**Published:** 2026-07-31

**Authors:** Andrew Kanouse, Michael McDonagh, Joanna S. Fishbein, Parissa Salemi

**Affiliations:** 1 Division of Diabetes and Endocrinology, Department of Pediatrics, Cohen Children’s Medical Center, Northwell Health, New Hyde Park, NY; 2 Biostatistics Unit, Office of Academic Affairs, Northwell Health, New Hyde Park, NY, United States

**Keywords:** autoimmunity, COVID-19 pandemic, HbA1c, pediatric diabetes, type 1 diabetes

## Abstract

**Background:**

Studies since the beginning of the COVID-19 pandemic suggest an increase in new cases of type 1 diabetes (T1D) but the etiology of this increase remains unclear.

**Objective:**

To examine whether there was a change in yearly proportions of newly diagnosed T1D by a single-center tertiary pediatric endocrinology practice in the post-pandemic era in comparison to pre-pandemic, T1D presenting characteristics, and the relationship of T1D antibodies both in those diagnosed before the pandemic versus those diagnosed after and those with COVID-19 versus other or no known respiratory viral illnesses.

**Methods:**

The study consisted of a retrospective chart review of children diagnosed with T1D between January 2018 and May 2022, stratifying them into cohorts based on respiratory infection status: known COVID-19 respiratory infection (COVID), other upper respiratory infection (URI), and no known viral respiratory infection (NONE). There was comparison of autoantibody positivity (type, number, and titer) between cohorts using non-parametric tests and descriptive statistics.

**Results:**

A total of 351 patients were included (mean age 9.8 ± 4.2 years; 59.4% male; 48.4% White, 18.2% African American, 8.3% Asian, 15.4% Hispanic/Latino, 24.8% other). At diagnosis, mean hemoglobin A1c (HbA1c) was 11.5 ± 2.3%. Overall T1D rates did not differ significantly between pre-pandemic (n=162, 46%) and post-pandemic (n=189, 54%) eras but new cases fluctuated significantly by year (*p* = 0.001). New annual cases peaked in 2020 (2.4%), representing a statistically significant increase from 2019 (1.4%, *p* = 0.0005) and a subsequent significant decline by 2022 (1.1%, *p* = 0.0006). Patients were stratified by infection history within 2 months of diagnosis: COVID (n=23, 6.6%), URI (n=53, 15.1%), and NONE (n=275, 78.4%). No significant differences were observed between these groups regarding body mass index, HbA1c, or diabetic ketoacidosis (DKA) severity. Among those with antibody data (n=236), positivity rates were 72.5% for zinc transporter antibody, 80.0% for glutamic acid decarboxylase, 51.5% for islet cell antibody, and 47.3% for islet antigen 2 antibody. There were no statistically significant differences in antibody titers or the number of positive antibodies between the disease cohorts or pre- and post-pandemic cohorts.

**Conclusions:**

These findings suggest that the COVID-19 pandemic may have accelerated disease progression in predisposed individuals rather than inducing T1D through COVID-19-mediated autoimmunity.

## 1 Introduction

Type 1 diabetes (T1D) is a complex disease characterized by insulin deficiency that affects approximately 1.5 million children worldwide, with prevalence continuing to rise 2-3% annually (1, 2). T1D is caused by autoimmunity against the pancreatic β-cells and, like other autoimmune diseases, occurs in individuals with a genetic predisposition (3). Viral illnesses have long been theorized as a triggering event for T1D (4). With the COVID-19 pandemic, research has increasingly focused on identifying epigenetic triggers that may precipitate disease onset in susceptible individuals contributing to the rise in T1D.

Declared a pandemic in March 2020 by the World Health Organization, COVID-19 resulted in significant morbidity and mortality, particularly among individuals with pre-existing diabetes (5, 6). While the interplay between severity of COVID-19 morbidity in someone with T1D became apparent early on, literature has emerged depicting a more bidirectional relationship where incidence data suggest the pandemic led to an increase in the number of new cases of T1D (7–10). A landmark report from the Centers for Disease Control and Prevention noted a significant rise in new diagnoses of diabetes (type 1 and type 2) in the first 15 months of the pandemic of up to 166% more in those with COVID-19 infection (11). What remained unclear at the time of this report was why this increase occurred and whether this would persist longitudinally.

Many studies have noted an acute increase in diabetes early in the pandemic but have not examined this long-term to determine if this trend persisted (5, 9, 10). The aim of this study was to evaluate the proportion of cases of new onset T1D during the peak of the COVID-19 pandemic compared to prior to the pandemic. Additionally, while many have hypothesized the increased incidence is secondary to an increase in COVID-19-induced autoimmunity, few studies have evaluated markers of diabetes autoimmunity to support this. With this objective in mind, an evaluation of autoantibody biomarkers was undertaken in both pre-pandemic and post-pandemic children and adolescents with new onset T1D. Additionally, this evaluation was stratified by whether individuals had a COVID-19 infection (cohort referred herein as: COVID), other upper respiratory infection (URI), or no known respiratory infection (NONE). The overall goal was to explore if an association exists between COVID-19 infection and autoimmunity as a link between the observed increase in incidence of T1D during the pandemic.

## 2 Methods

### 2.1 Study design and setting

This study consisted of a retrospective chart review of children and adolescents with new-onset T1D at a single-center tertiary pediatric endocrinology practice between January 2018 and May 2022. The clinic primarily serves Nassau and Queens Counties in New York. To validate the stability of the referral base, analysis was done of local census data for individuals under 18 years of age. The average population remained stable between the 2018–2019 and 2020–2022 periods (745, 939 versus 749, 695), indicating no significant change in the potential patient referral base (12).

### 2.2 Participant selection and eligibility

Patients were included if they were diagnosed with new-onset T1D based on clinical presentation and autoantibody status. The following cutoffs defined normal antibody values:

- Zinc Transporter 8 (ZT): < 15 U/mL; assessed via Enzyme-Linked Immunosorbent Assay (ELISA).
- Anti-insulin Antibody (IA): < 5.0 uU/mL; assessed via insulin-I125 binding capacity.
- Glutamic Acid Decarboxylase (GAD): 0.0 nmol/L; assessed via immunoprecipitation assay.
- Islet Cell Antibody (ICA) IgG ratio: < 1:4; assessed via semi-quantitative indirect fluorescent antibody.

Exclusion Criteria: Excluded were patients with more than one URI within the six-month period prior to T1D diagnosis. Additionally, one subject who tested positive for both COVID-19 and a URI was removed from the analysis.

### 2.3 Data collection and stratification

Baseline demographic and clinical data were extracted from medical records. The data were assessed for the presence of COVID-19 infection or other respiratory viral infections (URI) within the two months preceding or at the time of T1D diagnosis; this window was chosen to optimize recall accuracy. Infection history was determined via polymerase chain reaction (PCR) testing (if hospitalized), documented medical history, or patient self-report.

Subjects were stratified into three infection-status groups: COVID, URI, and NONE (either unknown, not reported, or known negative history). Furthermore, patients were categorized by diagnosis date relative to the pandemic:

- Pre-COVID-19: January 1, 2018 – February 28, 2020.
- Post-COVID-19: March 1, 2020 – May 19, 2022.

### 2.4 Clinical definition of diabetic ketoacidosis

DKA severity was defined as:

- Mild: pH 7.2–7.3 and/or serum bicarbonate 10–15 mmol/L.
- Moderate: pH 7.1–7.19 and/or serum bicarbonate 5–9.9 mmol/L.
- Severe: pH < 7.1 and/or serum bicarbonate < 5 mmol/L.

### 2.5 Statistical analysis

The proportion of new clinic patients of T1D was calculated as the number of new T1D cases divided by the total number of new patients seen at the clinic during the study. Proportion was used as a surrogate for incidence in this study given that the data analyzed new diagnoses in comparison to patients referred to the clinic of this catchment area.

Descriptive statistics included means (with standard deviation (SD)) or medians (with interquartile ranges (IQR)) for continuous variables and frequencies/proportions for categorical data. Categorical variables were compared using Chi-square or Fisher’s exact tests. Positive antibody status was computed for each antibody and a sum variable for the total number of positive antibodies was calculated (for IA, ICA and GAD). ZT antibody status was not included for inferential analysis (only descriptive) because of the large degree of missing data due to inconsistent autoantibody testing. Continuous variables were compared using Wilcoxon rank-sum or Kruskal-Wallis tests. Annualized proportions of new patients (2018, 2019, 2021, 2022) were compared to the 2020 rate with an adjusted *p*-value of 0.0125 for multiple comparisons. Since some subjects did not have complete antibody status (for IA, ICA, GAD), evaluation of patient demographics and illness category among subjects with missing data were compared to those with antibody data (for each antibody). No significant differences were found between missing and non-missing cases, so complete-case analysis was used for all analyses. Unless otherwise specified, level of significance was set at *p* < 0.05. A formal power calculation was not performed as the sample size was determined by the total clinic presentation during the study period. Analyses were performed using SAS software (v9.4) and visualized via GraphPad Prism (v10.4.1).

### 2.6 Ethical considerations

**2.** This study was approved by the Institutional Review Board of the Feinstein Institutes for Medical Research at Northwell Health.

## 3 Results

### 3.1 Data on proportion of new patients diagnosed with T1D

**Figure 1:**
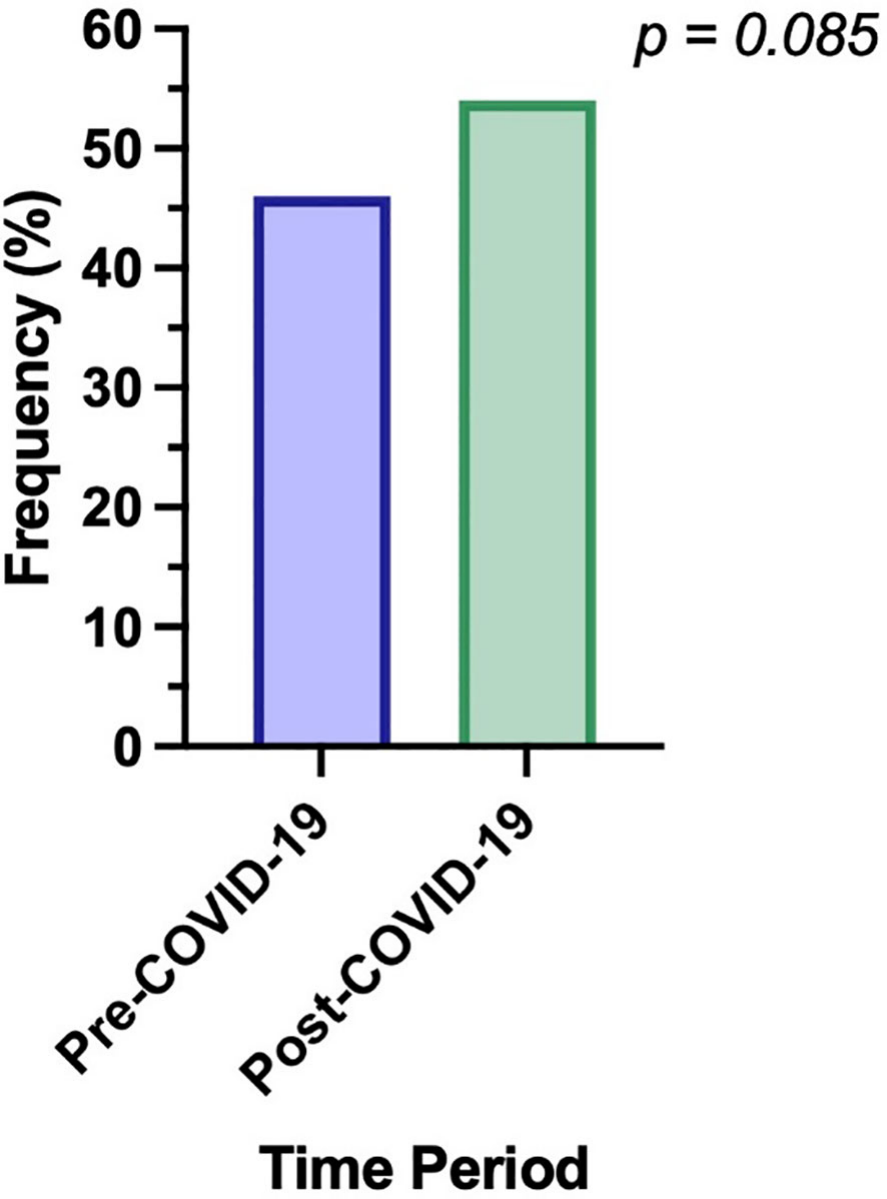
Proportion of new clinic patients diagnosed with T1D in relation to pandemic status.

**Figure 2:**
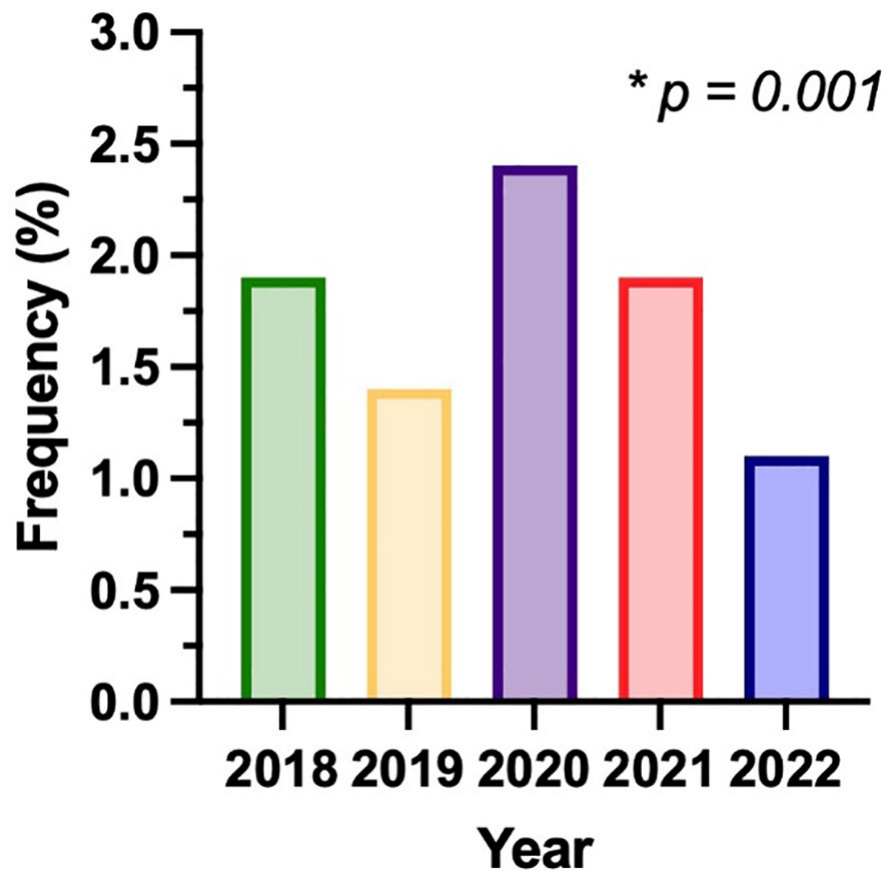
Proportion of new clinic patients diagnosed with T1D by year. *p=0.001.

A total of 351 individuals met inclusion criteria. There were 162 (46%) children diagnosed pre-pandemic and 189 (54%) during the pandemic (**Figure 1**). Although diagnosis rates were not significantly different between the two eras among new patients seen in the clinic, differences in annual newly diagnosed rates were found: 1.9% in 2018, 1.4% in 2019, 2.4% in 2020, 1.9% in 2021, and 1.1% in 2022. There was a statistically significant difference in proportion of newly diagnosed patients across years overall (*p* = 0.001). Results from the pairwise comparisons showed no significant difference between 2018 and 2020 rates or between 2020 and 2021 rates, but significant differences were observed between 2019 and 2020 (increase from 1.4% to 2.4%, respectively) and between 2020 and 2022 (decline from 2.4% to 1.1%) (*p* = 0.0005 and *p* = 0.0006, respectively). See **Figures 1**, **2**.

### 3.2 Demographic and clinical characteristics

The cohort was comprised of 209 (59.4%) males and 142 (40.5) females with the following racial/ethnic distribution: White (n=170, 48.4%), African American (AA; n=64, 18.2%), Asian (n=29, 8.3%), Hispanic/Latino (H/L; n=54, 15.4%), Native American (Native Amer; n= 1, 0.3%), and other ethnicity (n=87, 24.8%). Mean ± SD age was 9.8 ± 4.2 years, mean body mass index (BMI) percentile was 52.8 ± 35.1%, and mean initial hemoglobin A1c (HbA1c) was 11.5 ± 2.3%. See **Table 1**.

**Table 1:** Characteristics of newly diagnosed patients with type 1 diabetes stratified by infection status.

Characteristics	COVID	URI	NONE	*p*-value
Sex				
Female	14 (9.9%	17 (12.0%)	111 (78.2%)	*p* = 0.06
Male	9 (4.3%)	36 (17.2%)	164 (78.5%)	
Ethnicity				
White	11 (6.5%)	28 (16.5%)	131 (77.1%)	
AA	5 (7.8%)	5 (7.8%)	54 (84.4%)	
Asian	3 (10.3%)	7 (24.1%)	19 (65.5%)	*p* = 0.32
H/L	1 (1.9%)	9 (16.7%)	44 (81.5%)	
Native Amer^^^		13 (14.8%)		
Other	4 (4.6%)		71 (80.7%)	
Age (years)	10.3 ± 4.5	7.4 ± 4.3	10.2 ± 4.0	*p* = 0.0002
BMI (percentile)	55.5 ± 35.2	52.9 ± 38.1	52.5 ± 34.7	*p* = 0.98
Initial HgbA1c (%)	12.3 ± 2.5	11.3 ± 2.2	11.4 ± 2.3	*p* = 0.20
DKA				
None	8 (4.5%)	21 (11.9%)	147 (83.5%)	
Mild	5 (8.1%)	8 (12.9%)	49 (79.0%)	*p* = 0.17
Moderate	4 (7.5%)	11 (20.8%)	38 (71.7%)	
Severe	6 (10.0%)	13 (21.7%)	41 (68.3%)	
Initial Glucose (mg/dL)	434 ± 184	508 ± 219	461 ± 196	*p* = 0.96

*COVID-19 infection (COVID), other upper respiratory infection (URI), and no known respiratory infection (NONE).

**Variables are presented as frequencies (%) of the total cohort and then as frequencies (%) by each variable stratified by disease and mean ± SD.

***Native American data included in Other category for statistical analysis.

### 3.3 Cohort by disease status

Regarding respiratory infections, 23 (6.6%; 39.1% male) were in the COVID cohort, 53 (15.1%; 67.9% male) had a URI, and 275 (78.4%; 59.6% male) were in the NONE cohort in the 6 months at/prior to T1D diagnosis. DKA presentation at diagnosis was as follows: 176 (50.1%; 34.8% of the COVID cohort and 36.62% of the URI cohort) no DKA, 62 (17.7%; 21.7% COVID and 15.1% URI) mild DKA, 53 (15.1; 17.4% COVID and 20.8% URI) moderate DKA, and 60 (17.1%; 26.1% COVID and 24.5% URI) severe DKA. The observed mean age was lower in the URI group (7.4 ± 4.3 years), but appears comparable between the COVID and NONE groups (10.3 ± 4.5 and 10.2 ± 4.0, respectively; *p* = 0.0002. No significant differences were found between groups for BMI, initial HbA1c, pH, bicarbonate, or initial glucose.

### 3.4 T1D autoantibody status

**Figure 3:**
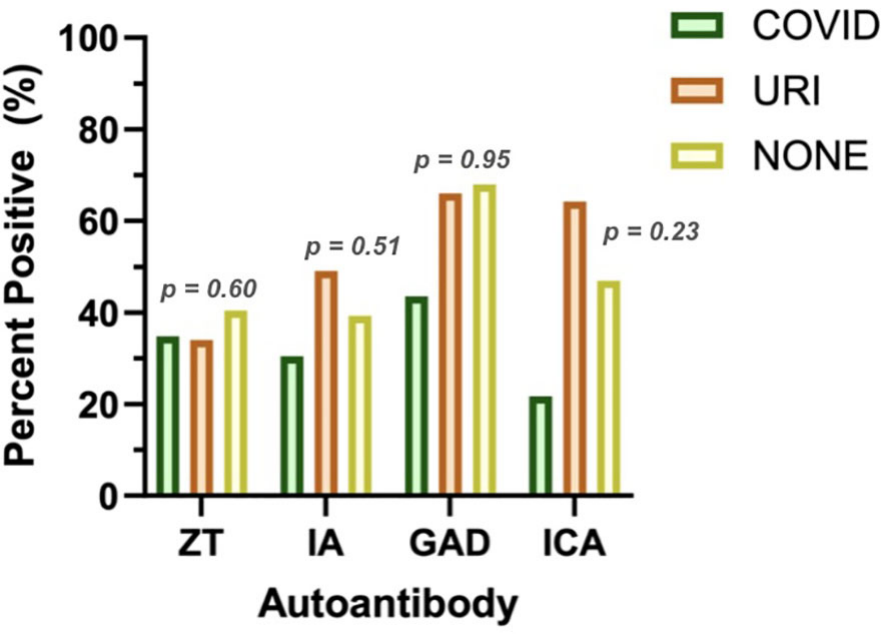
Distribution of type of T1D autoantibody positivity by infection status. Zinc transporter 8 antibody (ZT), insulin antibody (IA), glutamic acid decarboxylase antibody (GAD), islet cell antibody (ICA).

Complete antibody status (i.e. inclusion of IA, ICA, GAD) was available for 236 children (**Table 2**); Antibody evaluation did not occur equally amongst all individuals: 7.1% were missing ICA, 17.4% were missing GAD, 15.1% were missing IA and 46.2% were missing ZT. Among the subset of 236 with IA, ICA, and GAD autoantibody data, 137/189 (72.5%; 34.8% of the COVID cohort and 34.0% of the URI cohort; *p* = 0.60) had a positive ZT, 141/209 (47.3%; 30.4% COVID and 49.1% URI; *p* = 0.51) had a positive IA, 232/290 (80.0%; 43.5% COVID and 66.0% URI; *p* = 0.95) had a positive GAD, and 168/326 (51.5%; 21.7% COVID and 64.2% URI; *p* = 0.23) had a positive ICA. (**Figure 3**).

**Table 2:** Distribution of type of T1D autoantibody overall and by time period.

Antibody	Positive	Pre-COVID-19	Post-COVID-19	
ZT	137/189 (72.5%)	47 (34.3%)	90 (65.7%)	*p* = 0.48
IA	141/209 (47.3%)	70 (50.0%)	71 (50.4%)	*p* = 0.51
GAD	232/290 (80.0%)	119 (51.3%)	113 (48.7%)	*p* = 0.95
ICA	168/236 (51.5%)	74 (44.1%)	94 (56.0%)	*p* = 0.23

*Variables are presented as frequencies (%) of the total cohort and then as frequencies (%) by each variable stratified by disease.

^+^Zinc transporter 8 antibody (ZT), insulin antibody (IA), glutamic acid decarboxylase antibody (GAD), islet cell antibody (ICA).

**Figure 4:**
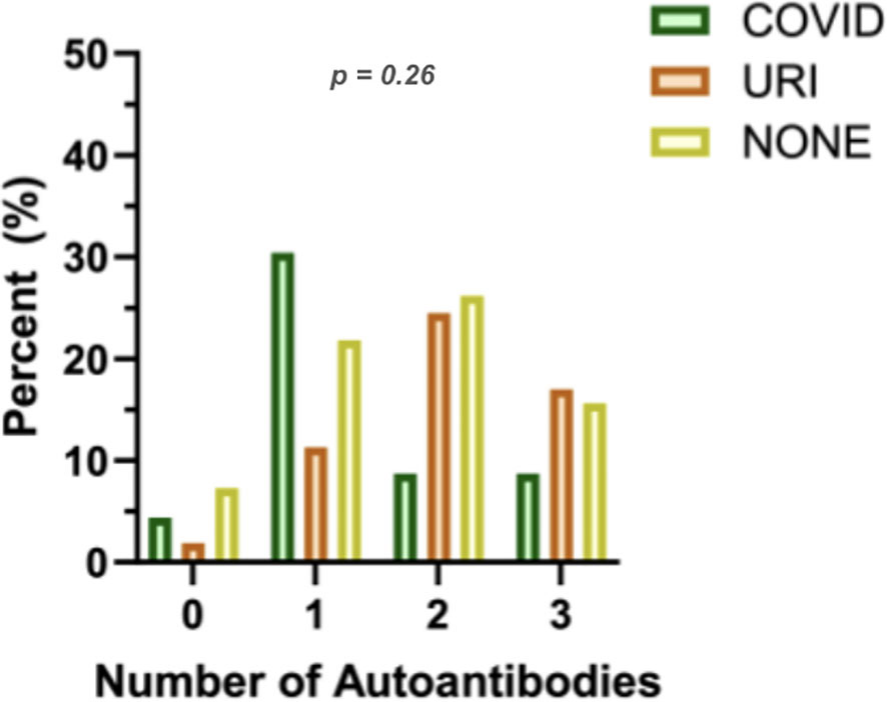
Distribution of number of T1D autoantibody positivity by infection status. ZT antibody not included in analysis of total antibody positive status because of the number in whom ZT was tested. The subset of 236 subjects with IA, GAD, and ICA autoantibody data were included in this analysis.

There were 73 (30.9%; 30.4% of the COVID cohort and 11.3% of the URI cohort) individuals with 1 positive antibody, 87 (36.9%; 8.7% COVID and 24.5% URI) with 2, 54 (22.9%; 8.7% COVID and 17% URI) with 3, and 22 (9.3%; 4.4% COVID and 1.9% URI) who had no positive antibodies. (**Figure 4**).

Median (IQR) values for antibodies as follows: 1) ZT 223 U/mL (0.0 - 501); 2) IA 0 uU/mL (0.0 - 3.3); 3) GAD 0.2 nmol/L (0.0 - 0.9); 4) ICA 1:20 (1:0 – 1:160). There was not a statistically significant difference in the level of antibody positivity between COVID, URI, and NONE cohorts (IA *p* = 0.32 and *p* = 0.29, GAD *p* = 0.65, and ICA *p* = 0.97). There was also not a significant difference in the level of antibody positivity pre- and post-pandemic (IA *p* = 0.07, GAD *p* = 0.67, and ICA *p* = 0.09).

## 4 Discussion

The interplay between genetic susceptibility and environmental exposures in T1D pathogenesis remains an area of active investigation, particularly regarding environmental triggers of clinical disease onset. Following the COVID-19 pandemic, multiple studies reported an increase in T1D incidence, raising concern for a diabetogenic role of the virus through induction of autoimmunity (10). However, findings regarding clinical presentation and proposed mechanisms remain inconsistent.

This study evaluated whether COVID-19 infection induces islet autoimmunity or whether data would suggest that indirect, pandemic-related factors contributed to the observed rise in new cases of T1D. Comparison of patients with new-onset T1D across pre- and post-pandemic periods, as well as among COVID-19, other respiratory viral infection, and no respiratory infection cohorts demonstrated no significant differences in autoantibody type, titer, or number of positive antibody. These findings contrast with hypothesized mechanisms implicating COVID-19 virus-mediated autoimmunity, including molecular mimicry, epitope spreading, neoepitope formation, stress-induced autoimmunity, or cytokine-driven β-cell destruction, and instead suggest alternative explanations for increased incidence (7, 8, 10, 13).

These data show a marked increase in the proportion of new cases of T1D that presented to clinic during the first year of the pandemic (2020), a period of stabilization in this proportion to pre-pandemic norms (2021), and a decline to below pre-pandemic levels (2022). The proportion of new T1D diagnoses increased from 1.7% in 2018–2019 to 2.4% in 2020, before decreasing to 1.8% in 2021 and 1.1% in 2022. While the average proportion of new cases across the pandemic period (2020-2022) was 1.9%, mirroring pre-pandemic levels, the initial spike suggests accelerated disease progression in genetically predisposed individuals rather than *de novo* T1D development. This interpretation is further supported by the similar antibody profiles across the various disease states and between pre- and post-pandemic periods. While not found in this study, Reitzle et al. examined the incidence of diabetes during the pandemic and observed an increase in T1D incidence driven by diagnoses in younger children in Germany, supporting the theory of accelerated progression to stage 3 (clinical disease) diabetes (7). While not statistically significant, the data herein also show an observed increase in the number of new diagnoses with only 1 positive T1D autoantibody in the COVID cohort, which would support the theory of a more rapid progression toward clinical disease. Importantly, prior studies that observed an increased incidence did not evaluate longitudinal trends to observe for a sustained increase or decline in incidence as this study does and thus cannot comment on an overall change in the trend of diagnoses.

Additional population-level studies support an acceleration hypothesis. McKeigue et al. observed a shortened interval from symptom onset to diagnosis and noted a mismatch between peaks in T1D incidence and COVID-19 infection rates in Scotland, disputing the disease itself as the initial cause (14, 15). Their examination of incident diabetes in Scotland found that an increase in new cases of T1D in their population occurred before the peak of COVID-19 infection rates and corresponded instead with peak in social distancing and non-infection, suggesting alternative factors that contributed to this increase in incidence (14). Blumenfeld et al. found a similar acute increase as the data presented here in incidence in 2020 and 2021 that had a steep decline in 2022, which they attribute to the timing of vaccine availability (4). They posit the hypothesis, supported by the data here, that those who developed T1D already had pre-existing β-cell dysfunction (incipient T1D) and that COVID-19 was the final inciting trigger for fulminant disease (4). Finally, a study by Bjerregaard-Andersen et al. in new diagnoses in Portugal observed a similar rise in cases specifically in 2020 and 2021 without changes in other metabolic patterns at diagnosis but with GAD antibody predominance (16). These findings are consistent with the temporal pattern observed in the cohorts presented here.

Pandemic-related environmental factors may better explain the observed acceleration to clinical disease. The hygiene hypothesis proposes that reduced microbial exposure during lockdowns impaired immune regulation, increasing susceptibility to autoimmune activation (9, 17, 18). For example, Liston et al. discuss the Karelia region of Finland/Russia, which has a rather homogenous genetic makeup but socioeconomically is divided into what they describe as variability between groups regarding prioritization of hygienic practices (17). They describe that the former cohort (more hygienic and so less viral infections) has a sixfold increase in incidence of T1D (17). In the context of the pandemic, this would suggest that the pandemic lockdown itself, not the COVID-19 virus, is the culprit of the increase in T1D incidence, which is supported by the data of this study. However, caution should be raised that this is a different study design in terms of region than the study presented herein. It is also postulated that the stress of the pandemic (social isolation, economic hardship, fear of mortality) accelerated the rate at which β-cell destruction occurred through an increase in cell fragility. This would be in line with why there was a noted increase in incident T1D following other stressful historic events, such as the Chernobyl nuclear meltdown and the 1994 Los Angeles earthquake (19). It is also well established that inflammation, be it from psychological stress, poorer metabolic statuses during the lockdown, or other environmental etiologies, also results in increased insulin resistance and thus increasing the burden on vulnerable β-cells, further hastening insulinopenia (20). Finally, pandemic-related microbiota dysbiosis could contribute. There is an association between less diverse fecal microbiota and development of islet autoimmunity so less biodiverse exposures as a result of the pandemic could have accelerated this connection and progression toward T1D (17, 18).

Pediatric studies examining antibody positivity in the context of increased pandemic T1D incidence remain limited. To our knowledge, there are no known studies comparing the COVID-19 virus with other respiratory viruses or no respiratory disease in terms of antibody positivity. Additionally, minimal studies have evaluated islet cell biomarkers between pre- and post-pandemic periods, ultimately questioning if there is an autoimmune connection between COVID-19 and development of T1D. This study did not find a difference in autoimmune biomarkers, demonstrating similar antibody profiles between both time periods and disease group despite an initial increase in T1D incidence. This concurs with the findings presented in a study of 51, 970 healthy children in the United States and Germany that found 4, 386 had COVID-19 antibodies, but that there was not an association found between this and the presence of pancreatic islet autoantibodies (21). Margolis et al. reached a similar conclusion in their evaluation of new diagnoses before and after the pandemic, finding, like in this study, no significant difference in pancreatic autoantibody positivity and antibody titer, but still a rise in new diagnoses in the first two years of the pandemic suggesting a role of non-autoimmunity (22). Their study had a statistically significant observation that diagnoses were more likely to have multiple positive autoantibodies prior to the pandemic (22). They did not, however, compare this in relation to changes in proportions of new diagnoses or in comparison to other respiratory viruses.

Finally, Knip et al. in Finland not only corroborate findings in their cohort that the peak of COVID-19 disease and new onset T1D is discrepant and therefore the virus is unlikely to have caused this increase, but they also found that the only change in autoantibody positivity was an increase in GAD in those diagnosed during the pandemic (23). They postulate that, as GAD is associated with slower progression to clinical disease, this indicates that individuals more likely to have had slowly progressive disease had clinical disease during the pandemic, in line with the acceleration theory outlined prior (23). In contradiction, a smaller study by Boboc et al. found higher increased islet antigen 2 (IA2) antibody (not assessed in this study) positivity in children found to be positive for COVID-19 infection (24). However, such findings still cannot determine causality and whether the virus induced these antibodies independently or accelerated the timing of when they would have naturally developed. It is possible that the lack of difference between autoantibody positivity is a reflection that these antibodies are not the direct cause of the T1D, but rather biomarkers of disease, and that there remains a pathway that has yet to be elucidated that is measured through a different biomarker explaining the connection between either the virus or pandemic and T1D development (20).

Key limitations of this study include the relatively small COVID-19 cohort, potential misclassification of infection status, and selection bias inherent to its retrospective design, raising the potential for a type II error statistically. Undiagnosed prior COVID-19 infection in the NONE group and false-negative PCR testing at diagnosis cannot be excluded. Self-reported infection history introduces recall bias and presence of ascertainment bias given that clinic presentation patterns varied during the pandemic may affect generalizability. Additionally, incomplete autoantibody panels, including lack of IA2 testing, and lack of assessment of vaccination status limit interpretation. Regarding the former, although missing data were described, formal imputation or inverse probability weighting was not performed thus limiting analysis to those with complete data, which may introduce bias and affect generalizability. The younger age of children diagnosed in the URI cohort may reflect distinct viral influences on T1D pathogenesis that differ from COVID-19–related mechanisms. It is also possible that such fluctuations continued throughout the pandemic and further investigation may be warranted for further into this period to evaluate such. For example, the acceleration hypothesis may appear to be temporary within the 2020–2022 period and that follow-up studies beyond 2022 are needed to confirm the hypothesis beyond. Location may also be considered a limitation, as this study was conducted in a single center in New York, where lockdown and pandemic measures/public responses may differ from other cities thus limiting the generalizability of this hygiene hypothesis’ influence globally on the pathogenesis of T1D. While not standard of care for diagnosis of new-onset diabetes, this study data does not include measurement of c-peptide levels and thus cannot confirm acceleration of pancreatic dysfunction. Finally, although the possibility remains that referral patterns may have shifted during this period, with some centers receiving fewer referrals, Cohen Children’s Medical Center—the only freestanding children’s hospital in Nassau and Queens counties—remains the primary destination for pediatric subspecialty referrals in this region. As a result, it is unlikely that referral volumes would have significantly declined in subsequent years at this clinic.

## 5 Conclusions

T1D is a lifelong, multifaceted disease with a profound impact on daily life. While the precise mechanisms linking genetic predisposition and clinical disease remain unclear, understanding potential risk factors is crucial for informing at-risk individuals. The observed increase in T1D cases presenting to our clinic following the COVID-19 pandemic raised concerns about the virus directly inducing T1D through increased autoimmunity. However, this data suggest that the pandemic may have accelerated disease progression in genetically predisposed individuals, rather than triggering T1D *de novo*. Despite the study’s limitations, these findings may inform risk assessment in future pandemics or other stressful global phenomena and heighten the need for awareness of more rigorous screening for symptoms of incipient diabetes.

## Data Availability

The raw data supporting the conclusions of this article will be made available by the authors, without undue reservation.
